# The effect of whole-body electromyostimulation on metabolic syndrome in affected adults: A subanalysis of a randomized controlled trial

**DOI:** 10.3389/fspor.2025.1585579

**Published:** 2025-07-01

**Authors:** Leon Mendel, Stephanie Kast, Simon von Stengel, Matthias Kohl, Frank W. Roemer, Michael Uder, Wolfgang Kemmler

**Affiliations:** ^1^ Institute of Radiology, University Hospital Erlangen, Erlangen, Germany; ^2^ Faculty of Health, Medical and Life Sciences, University of Furtwangen, Schwenningen, Germany; ^3^ Department of Radiology, Chobanian & Avedisian School of Medicine, Boston University, Boston, MA, United States

**Keywords:** metabolic syndrome, overweight, obesity, whole-body electromyostimulation, cardiometabolic risk factors

## Abstract

Many people with osteoarthritis of the knee suffer from overweight, obesity, and cardiometabolic conditions. In the present subanalysis of a randomized controlled trial of the effect of whole-body electromyostimulation (WB-EMS) on knee osteoarthritis in overweight Caucasians, we focus on participants with Metabolic Syndrome (MetS). Based on previous research, we hypothesized that WB-EMS significantly improves the Metabolic Syndrome *Z* score (MetS-Z score) compared with non-training controls. Thirty-two of the initial 72 overweight adults (58 ± 6 years, body mass index: 31 ± 4 kg/m^2^) with knee osteoarthritis, randomly allocated to a 29-week standard WB-EMS application or to a non-exercising control group (CG) and suffering from MetS, were included. The primary outcome was the MetS-Z score, based on the criteria of the International Diabetes Federation. Secondary outcomes were MetS components, i.e., waist circumference, mean arterial blood pressure, fasting glucose, triglycerides, and HDL-cholesterol. Based on the intention-to-treat principle, analysis of covariance determines differences between the groups (i.e., “effects”). In total, three participants were lost to 29-week follow-up. The attendance rate averaged 89% ± 9% in the WB-EMS group. Adverse effects related to the intervention were not observed. WB-EMS (*n* = 17) induced a non-significant, medium-size effect (*p* = 0.061; *η*^2^ = 0.13) on the MetS-Z score compared with non-exercise CG (*n* = 15). In addition, no significant effects (*p* ≥ 0.146) were observed for MetS components. In the present study, we observed a moderate, although non-significant effect on the MetS-Z score. Given that the WB-EMS application was well-tolerated and accepted by the participants, we conclude that this exercise technology may offer (limited) benefits for MetS treatment. Nevertheless, further studies should address this issue with higher statistical power.

## Introduction

There is consensus that physical activity and exercise positively impact cardiometabolic conditions (1). However, due to musculoskeletal and/or cardiovascular diseases, many people are unable or limited in their ability to conduct exercise with intensity levels adequate to address health-related outcomes (2). People with osteoarthritis (OA) might be such a cohort. Considering the close association of knee OA and overweight/obesity not only due to higher mechanical load but also to the proinflammatory effects of increased visceral adipose tissue (VAT) fraction (3), people with knee OA are at increased risk of cardiometabolic diseases (4, 5). Because of its time efficiency and joint friendliness, whole-body electromyostimulation (WB-EMS) is a safe and attractive training technology for people with limited options for conventional exercise (6). Although there is some evidence that WB-EMS provides similar positive changes on cardiometabolic outcomes as high-intensity resistance exercise (7), only a few studies have focused on WB-EMS effects on cardiometabolic outcomes (8). Recently, a systematic review and meta-analysis summarized the effect of WB-EMS on Metabolic Syndrome (MetS) (9). Although the authors observed a small but significant effect and reported low heterogeneity in trial results, cohorts and WB-EMS protocols of the individual studies vary considerably. This includes (superimposed) WB-EMS protocols not or not adequately suitable for people with OA of the lower extremities. This led us to determine the effects of WB-EMS on MetS in overweight to obese people with OA of the knee. Our primary hypothesis was that standard WB-EMS application (8) significantly improves MetS as summarized in a continuous Z score, which combined individual values and cut-offs for the five MetS components (10), compared with a non-training control group (CG) with usual (OA) care in people with knee OA and the MetS according to the International Diabetes Federation (IDF) (11).

## Material and methods

### Study design

The present study is part of the randomized controlled “electromyostimulation for the treatment of knee osteoarthritis (OA) (EMSOAT) study” (12), a WB-EMS trial that focuses on overweight and obese adults. However, in the present analysis, we focus on WB-EMS effects on MetS. EMSOAT was planned, initiated, and conducted by the Institute of Radiology, University Hospital Erlangen, Germany. The study was approved by the University Ethics Committee (Nr. 352_20 B) and was conducted in full adherence to the Helsinki Declaration (13). After detailed information, all study participants provided written approval. The project was registered under ClinicalTrials.gov (NCT05672264).

### Participants

**Figure 1:**
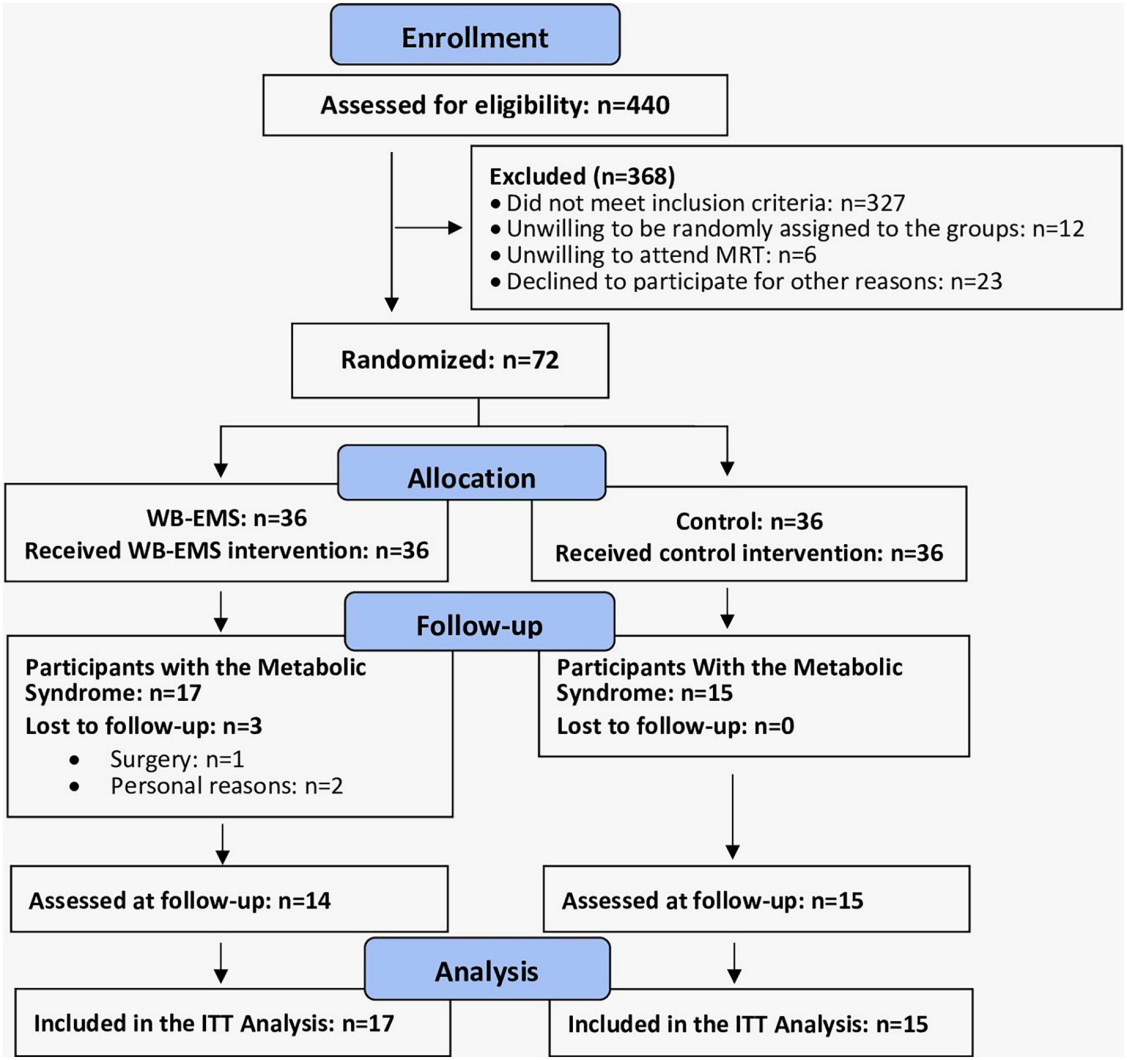
Participant flow through the study according to PRISMA.

The recruitment process of EMSOAT was described in detail in another publication (12). Briefly, participants were included by the study physician if they met the following inclusion criteria (Figure 1): (a) age 40–70 years, (b) overweight/obesity [body mass index (BMI) > 25 kg/m^2^], (c) knee OA Kellgren–Lawrence grades 2 and 3. Exclusion criteria were (a) WB-EMS or resistance exercise ≥60 min/week in the last 12 months; (b) glucocorticoid or opioid therapy; (c) trauma of the knee joint; (d) intra-articular knee injections in the last 12 weeks; (e) conditions, diseases, and corresponding therapy with relevant impact on our study outcomes (e.g., rheumatoid arthritis, fibromyalgia); and (f) contraindications for WB-EMS (14). In summary, a total of 72 participants were eligible and willing to participate and were randomly allocated to the WB-EMS (*n* = 36) and CG (*n* = 36). After adding the eligibility criteria of MetS according to the IDF (11) for this cohort, 17 participants of the WB-EMS and 15 participants of the CG were included in the present analysis.

### Randomization and blinding

Using the envelope method, lots were placed in small opaque capsules (“kinder egg”, Ferrero, Italy) and drawn from a bowl by the participants. A researcher not involved in the present project prepared the lots and supervised the procedure to realize allocation concealment. After the randomization procedure, the primary investigator (SK) enrolled participants and instructed them in detail about their study status and corresponding dos and don’ts. Outcome assessors were blinded to the participants’ group status (WB-EMS or CG) and were not allowed to ask.

### Study procedures

The WB-EMS group conducted the procedure described below, while the CG received a “usual care” intervention (physiotherapy) that can be considered ineffective in the context of the present research issue. In addition, both groups were asked to conduct a self-management education program for knee OA. Of importance, adherence to the WB-EMS protocol was closely monitored by the trainers. In parallel, adherence to physiotherapy was evident from the billing of physiotherapy practices.

### WB-EMS intervention

Briefly, we applied a consistently and closely supervised (one trainer—two trainees for each session) WB-EMS protocol (15) of 1.5 × 20 min/week (i.e., three sessions in 2 weeks) for 29 weeks. Both thighs and upper arms, gluteal, abdomen, chest, lower back, latissimus, and upper back were simultaneously stimulated by a miha bodytec®, Type II WB-EMS device (Gersthofen, Germany). We applied bipolar current with 85 Hz, 350 µs impulse width, and a direct impulse boost using an interval protocol with a 6-s impulse and 4-s impulse break. Impulse intensity was prescribed at “6-7” (i.e., “hard + to very hard”) on the Borg CR10 Scale. During the impulse phase, low-amplitude, low-intensity movements [e.g., low-amplitude squat with latissimus pulleys, butterfly reverse, straight pullovers with trunk flexion, one-legged stand (extended knee) with biceps curl] (16) were performed in a standing position.^1^ This protocol can be considered the standard protocol predominately applied in research and commercial facilities (8).

### Control intervention (physiotherapy)

Based on the underlying research topic of the EMSOAT project (knee OA) and in accordance with the German S2-guideline on knee OA, the CG underwent six physiotherapy sessions. To ensure that all participants of the CG received this “usual care” standard treatment, participants received a prescription for six physiotherapy treatment sessions (20 min each), prescribed by the study physician. The six sessions were carried out once a week during the first 2–3 months of the study. The treatment was carried out individually in a diagnosis-oriented manner. In summary, techniques to reduce pain and detonate the muscle tissue were applied. Further, in many cases, the physiotherapists aimed to improve knee joint mobility and leg muscle strength. Overall, the physiotherapeutic intervention of the EMSOAT project can largely be regarded as a non-training control group in the context of the present research question, particularly due to its non-specific content.

### Self-management education program for knee OA

A self-management program of osteoarthritis (17) with six sessions of 60 min each was applied for the WB-EMS and CG. Briefly, the program aimed to provide education, information, and counseling to prevent the progression of OA, reduce fear and avoidance attitudes, and thus improve the participants’ quality of life and mobility.

### Study outcomes

As described above, the EMSOAT study primarily concentrated on assessing outcomes linked to knee osteoarthritis. However, in this article, we focus on the MetS-Z score (10) based on the IDF-criteria (11), which includes waist circumference (WC), mean arterial blood pressure (MAP), fasting glucose (FPG), triglycerides (TG), and HDL-cholesterol (HDL-C) as components.

### Metabolic syndrome Z score (MetS-Z score)

• Changes in the MetS-Z score from baseline and 29-week follow-up (FU).

### Metabolic syndrome components

• Changes in waist circumference between baseline and 29-week FU.
• Changes in MAP between baseline and 29-week FU.
• Changes in FPG between baseline and 29-week FU.
• Changes in triglycerides between baseline and 29-week FU.
• Changes in HDL-cholesterol between baseline and 29-week FU.

### Assessments

#### Metabolic syndrome-Z score

We calculated a *Z* score using individual subject data, IDF MetS cut-off criteria (11), and standard deviations (SD; denominators of each factor in the formula) of the given (either men or women) cohort at baseline.

For the female participants, the MetS-Z score was calculated [(50 − HDL-C)/SD − HDL-C] + [(Triglycerides − 150)/SD − TGs] + [(fasting glucose − 100)/SD − FPG] + [(waist circumference − 88)/SD − WC] + [(mean arterial blood pressure − 100)/SD − MAP].

The corresponding MetS-Z score for the male participants was calculated [(40 − HDL-C)/SD − HDL-C] + [(triglycerides − 150)/SD − TGs] + [(fasting glucose − 100)/SD − FPG] + [(waist circumference − 80)/SD − WC] + [(mean arterial blood pressure − 100)/SD − MAP].

#### Metabolic syndrome components

##### Waist circumference

Waist circumference was determined in a standing position as the minimum circumference between the distal end of the rib cage and the top of the iliac crest along the midaxillary line at the end of a normal expiration.

##### Mean arterial blood pressure

After 10 min of relaxation, blood pressure was determined twice on the right arm with a rest of 20 s between the samples and in a sitting position. MAP was calculated using the formula (diastolic blood pressure + diastolic blood pressure + systolic blood pressure)/3.

##### Glucose, triglycerides, and HDL-C

After an overnight fast, blood was consistently sampled between 7:00 and 9:00 am in a sitting position from an antecubital vein. Serum samples were centrifuged for 20 min at 3,000 RPM and immediately analyzed. All biomarkers were measured in standard clinical laboratories.

### Baseline characteristics and confounding factors

Body height was measured using a Holtain stadiometer (Crymych Dyfed, UK) and body mass and body composition were determined by performing direct-segmental, multifrequency bioimpedance analysis (DSM-BIA, InBody 770, Seoul, Korea).

A standardized questionnaire gathered information on (a) demographic factors; (b) physical limitations, comorbidities, history of operations, pharmacological treatment, and dietary supplements; as well as (c) lifestyle aspects such as physical activity, exercise, and diet. After 29 weeks of intervention, participants were asked to complete the FU questionnaire that aimed to identify changes in conditions/diseases, pharmacologic and physical therapy, and exercise and diet, i.e., factors that could potentially affect the present study outcomes. Questionnaires were checked by the study physician together with the participant at 29-week FU to verify potential changes in confounding factors.

### Adverse effects of the interventions

Adverse effects (AEs) were defined as any untoward medical occurrence, disease or injury, or any untoward clinical signs, including an abnormal laboratory finding. In detail, AE monitoring focused predominately on the WB-EMS intervention and was conducted by WB-EMS trainers on a weekly basis. Apart from personal interviews, we determined inflammatory markers (e.g., ultrasensitive C-reactive protein, interleukin-1β) at baseline and 29-week FU (18).

### Sample size calculation

Sample size calculation [analysis of covariance (ANCOVA)] was based on the primary outcome of the EMSOAT project “pain of the knee joint” not addressed in the present contribution.

### Statistical analysis

We applied the intention-to-treat (ITT) principle with multiple imputation (R Development Core Team, Vienna, Austria) together with Amelia II (19) and analyzed all the participants who were randomly assigned to the study groups at the start of the study. Linear mixed-effects ANOVA was applied to analyze the effects of continuous within- and between-subjects variables. Pearson chi-square tests were used to analyze categorical variables (Table 1). Partial eta squared (*η*^2^) were used to indicate standardized effects sizes. All tests were two-tailed, and significance was accepted at *p* < 0.05.

**Table 1:** Baseline characteristics of the study participants.

Variable	CG (*n* = 15) MV ± SD	WB-EMS (*n* = 17) MV ± SD	*p*
Gender (women/men) (n)	13/2	11/6	0.152
Age (years)	57.5 ± 6.8	58.2 ± 5.7	0.753
Body height (cm)	171.5 ± 6.3	172.3 ± 9.2	0.773
Body mass (kg)^a^	87.3 ± 11.3	95.3 ± 15.8	0.100
Body mass index (kg/m^2^)	29.6 ± 3.5	32.1 ± 4.8	0.098
LBM (kg)^a^	53.5 ± 8.1	59.5 ± 13.5	0.148
Total body fat (%)^a^	38.4 ± 5.9	37.5 ± 9.5	0.774
Pain (Numeric Rating Scale 1–10) (n)	4.7 ± 2.0	4.0 ± 1.3	0.256
Physical activity (Index)^b^^,^^c^	3.7 ± 1.4	3.5 ± 1.2	0.755
Exercise volume (min/week)^c^	133 ± 103	156 ± 136	0.676
Number of diseases (n)^c^	1.5 ± 1.3	1.3 ± 1.0	0.569
Hypertension medication (n)^c^	3	4	0.801
Cholesterol-lowering drugs (n)	2	3	0.737
Analgesics (n)	8	8	0.723

MV, mean value; SD, standard deviation.

^a^ As assessed by BIA.

^b^ Index from 1: very low to 7: very high.

^c^ As determined by questionnaire and personal interview.

## Results

Three participants of the WB-EMS group were lost to follow-up. One participant was unable to visit the 29-week follow-up assessment due to surgery, and two participants of the WB-EMS group quit the study for personal reasons not related to the intervention (Figure 1). The attendance rate averaged 89% ± 9% in the WB-EMS group and more than 90% in the CG. No unintended adverse effects or injuries related to the WB-EMS application were observed, reported by the participants, or determined by laboratory findings (18).

Table 1 displays baseline results of the study cohort. In summary, no significant differences were observed between the WB-EMS and the CG at baseline. Nevertheless, differences for BMI and lean body mass (LBM) were quite pronounced, while body fat (%) was comparable between the groups. Apart from seven to five participants, respectively, who took medication for hypertension or hypercholesterinemia (Table 1), no further pharmacologic therapy (e.g., GLP-1 RA) that might have relevantly affected the present outcome was reported.

### Study outcomes

Table 2 shows the results for the MetS-Z score and its components at baseline along with changes after 29 weeks of intervention. Although the change in MetS-Z score did not reach statistical significance (*p* = 0.061), the effect size was moderate (*η*^2^ = 0.13), suggesting a potential positive trend. No significant between-group differences for prepost changes within the WB-EMS vs. CG were observed for the MetS components (Table 2). Further, significant pre- vs. postintervention changes within the groups were not observed for any of the study outcomes listed in Table 2.

**Table 2:** Baseline data and changes of the MetS-Z score and its components in the CG (*n* = 15) and WB-EMS group (*n* = 17) with corresponding between-group differences (linear mixed-effects ANOVA model).

Variable	CG MV ± SD	WB-EMS MV ± SD	Adjusted difference MV (95% CI)	*p*-value	Partial *η*^2^
MetS-Z score (index)					
Baseline	1.57 ± 3.02	1.38 ± 3.81	—	0.866	—
29-week FU	1.99 ± 2.08	0.62 ± 3.35	—	0.226	—
Changes	0.42 ± 1.60	−0.76 ± 1.83	−1.21 (−2.45 to 0.03)	0.061	0.13
Waist circumference					
Baseline	102.5 ± 7.9	104.4 ± 11.7	—	0.610	—
29-week FU	102.8 ± 7.8	104.8 ± 12.3	—	0.600	—
Changes	0.33 ± 2.90	0.42 ± 3.06	−0.06 (−2.08 to 2.21)	0.955	0.004
MAP (mmHg)					
Baseline	106.2 ± 11.2	109.2 ± 8.4	—	0.367	—
29-week FU	110.3 ± 7.8	107.0 ± 9.0	—	0.350	—
Changes	4.1 ± 11.6	−2.2 ± 12.4	−6.3 (−14.8 to 2.1)	0.146	0.07
FPG (mg/dl)					
Baseline	111.4 ± 30.5	101.1 ± 21.3	—	0.336	—
29-week FU	114.3 ± 39.8	95.9 ± 22.5	—	0.099	—
Changes	2.9 ± 21.0	−5.2 ± 26.8	−8.4 (−24.8 to 8.1)	0.323	0.05
Triglycerides (mg/dl)					
Baseline	135.5 ± 53.7	142.3 ± 94.1	—	0.831	—
29-week FU	145.6 ± 58.9	147.9 ± 98.6	—	0.926	—
Changes	10.1 ± 47.6	5.6 ± 60.6	−3.7 (−40.7 to 33.4)	0.847	0.008
HDL-cholesterol (mg/dl)					
Baseline	56.8 ± 7.8	55.4 ± 16.1	—	0.752	—
29-week FU	57.2 ± 6.3	58.6 ± 15.5	—	0.789	—
Changes	0.4 ± 5.8	3.1 ± 6.6	2.5 (−2.0 to 7.0)	0.285	0.06

MV, mean value; SD, standard deviation.

η^2^ = 0.01 indicates a small effect, η^2^ = 0.06 indicates a medium effect, and η^2^ = 0.14 indicates a large effect.

### Confounding factors

No significant changes within or between the groups were recorded for physical activity and exercise during the study period. Changes in dietary habits with lower carbohydrates/sugar and/or energy intake were reported by two participants of the WB-EMS and CG each, respectively. Antihypertensive medication was discontinued by one participant of the WB-EMS group, whereas changes in medication that focus on lipoproteins or blood lipids were not reported. In parallel, no conditions (e.g., eating disorders) or diseases (e.g., thyroid function) with a potential impact on the outcomes addressed here were recorded.

## Discussion

The present study addresses WB-EMS-induced effects on MetS and its components in a cohort of overweight to obese people with knee OA and MetS according to IDF (11). In summary, we have to reject our hypothesis of significant positive effects of a standard WB-EMS protocol (8) vs. a non-training CG on the MetS-Z score that summarized MetS components to a continuous score more sensitive to metabolic changes compared with the dichotomous MetS Index used for clinical purposes (10). We observed a non-significant WB-EMS effect of moderate size for the main study outcome MetS-Z score. Significant effects or group changes were not determined for any of the five MetS components. The limited benefit on the MetS-Z score and individual MetS components along with the finding that only one participant of the WB-EMS group lost his MetS status,^2^ however, raises the question of the clinical relevance of our results. So far, we estimate the clinical relevance of WB-EMS as an exercise therapy to fight the MetS in overweight to obese people suffering from the MetS to be low to moderate. On the other hand, potentially more effective types of training (e.g., weight-bearing endurance exercises) are often not applicable for people with knee OA. Furthermore, the current WB-EMS approach can be considered safe, well-tolerated, and (as proved by a high attendance rate) attractive for people with MetS.

A review of the literature may provide further insight into the clinical relevance of WB-EMS in combating the MetS. In a recent meta-analysis, Guretzki et al. (9) reported low but significant effects (d` = 0.33, *p* = 0.013) of WB-EMS on the MetS-Z score; however, the five available trials (7, 20–23) differ considerably with respect to cohort and WB-EMS application, thus preventing a comprehensive recommendation of WB-EMS to fight the MetS. Only two trials focus on people with established MetS (21, 22). In their 12-week pilot study with obese women 18 years and older with the MetS according to NCEP-ATP II^3^ criteria (24), Reljic et al. (21) reported significant MetS-Z score reductions in their WB-EMS group (2 × 20 min/week, *n* = 15) but no significant effects vs. their non-training CG (*n* = 14). In parallel, the same research group (22) reported no significant effects after 12 weeks of WB-EMS (2 × 20 min/week) in their obese MetS (NCEP-ATP III) patients (*n* = 20) undergoing caloric restriction, compared with corresponding non-exercise controls (*n* = 22). In contrast, Wittmann et al. (23), who applied WB-EMS (1 × 20 min/week) and protein supplementation for 6 months in older women with sarcopenic obesity (*n* = 25), observed significant effects on the MetS (NCEP-ATP III) compared with non-intervention control. However, no significant effect on the MetS-Z score was determined for the isolated WB-EMS intervention (23). The two remaining WB-EMS studies either applied superimposed WB-EMS [i.e., high-intensity interval training (HIIT) superimposed by WB-EMS] for 12 weeks (20)^4^ or compared their 16-week WB-EMS intervention with a similar time-effective high-intensity resistance exercise training (HIT-RT) (7)^5^ and were thus unable to determine the proper effect of (isolated) WB-EMS on MetS. To overcome these limitations of superimposed WB-EMS application (20), exercising control (7, 20), short intervention (20–22), or non-affected cohorts (7, 20, 23), the present study compared a standard (i.e., non-superimposed) 29-week WB-EMS protocol with a non-training CG with minor physical intervention in the relevant cohort of people with the MetS.

Although predominately cohorts with low affinity to conventional exercise training were attracted due to the specific character of WB-EMS and thus a comparison of WB-EMS effects on MetS with other types of exercise might be moot, we would like to shortly address this issue. Of note, in its present application with low training volume (1-2 × 20 min week) and intermittent bouts (4–6 s) of moderate to high stimulus intensity, WB-EMS can be considered a resistance-type exercise (8). As stated above, one study that compared WB-EMS with a single set resistance exercise training to muscular failure applying intensifying [i.e., HIT-RT (25)] and similarly time-effective strategies compared with WB-EMS (30 vs. 20 min) revealed non-significantly more favorable results on the MetS-Z score in favor of the WB-EMS intervention. Addressing endurance-type exercise, in contrast to older data (26), a more recent network meta-analysis (27) reported more favorable MetS changes after resistance compared with endurance exercise. In summary, the authors suggested combined resistance and endurance exercise programs as the most effective intervention for improving MetS and cardiovascular risk factors. WB-EMS has been applied by a few intervention studies as an endurance-type exercise, i.e., with consistent or longer impulse phases and lower impulse intensity superimposed by running or cycling exercise (20, 28). However, as already mentioned, superimposed (HIIT) endurance and WB-EMS were not superior to isolated HIIT protocols, and thus the combination of conventional endurance training and WB-EMS sessions might be a more feasible concept for addressing the MetS.

### Limitations and particularities of the study

While the results of the present study provide limited support for the application of WB-EMS in people with MetS, some limitations of the present trial should be considered by the reader. The present study reported the results of a subanalysis of a larger trial with overweight to obese people with knee OA. Thus, the sample size analysis does not focus on the present study outcome and randomization did not cover the present MetS cohort. Applying the data of the meta-analysis of Guretzki et al. (9) mentioned above, the present study would be underpowered. However considering the pronounced effect-lowering methodological limitations of most of the included studies,^6^ we simply expect higher effects and lower variations from the present study that compared WB-EMS vs. non-training control (see below).

We applied a standard WB-EMS protocol predominately applied in the nearly 2,000 commercial WB-EMS providers in Germany (8). Briefly, this includes the application of low-volume, non-superimposed, and consistently supervised WB-EMS with low impulse frequency (85 Hz) and an intermittent impulse protocol.

The EMSOAT project implemented a “usual care” (physiotherapy) CG that focused on a few physiotherapy sessions addressing OA during the first 3 months of the 29-week intervention. Considering the low-volume, intensity, and in particular the non-specificity of this intervention in the present context of MetS, the CG can be widely considered a “non-training” control group. Another issue that frequently arises is whether the movement component of WB-EMS should not (also) be performed by a (control) group with the identical movements without EMS application. This should be indeed the case in superimposed WB-EMS protocols; however, in the present study, only slight movements (not exercises) were applied during the impulse phase of the WB-EMS application, which *per se* relevantly affected (at least) musculoskeletal outcomes or biomarkers (29).

We applied the MetS-Z-score for several reasons. First of all, for the assessment of an intervention effect, a continuous score is more sensitive compared with the dichotomous categorization used for clinical purposes. Weighing the changes in each component based on their variance from healthy norms enables us to effectively monitor and predict shifts in cardiometabolic health status. In addition, individual components of MetS interact in complex ways. Addressing one component may, therefore, result in improvements across multiple factors, which cumulatively leads to a greater shift in the *Z* score.

One may argue that changes in cardiometabolic medications might have impacted our result. However, only one participant of the WB-EMS group reported termination of antihypertensive medication, while other changes of medication related to cardiometabolic conditions were not reported. Since in parallel no relevant changes of lifestyle, including exercise habits and diet, or upcoming conditions or diseases associated with the MetS were listed, we feel that it is justified to predominately attribute the favorable effect on the MetS to the WB-EMS intervention. Nevertheless, a more rigorous tracking of confounding factors including medication, physical activity, and diet should be applied in future studies.

Because of the limited sample size of the study, the analysis was not stratified for gender or age. However, a raw comparison did not indicate relevant sex or age effects. Nevertheless, it is difficult to generalize our results to other cohorts.

## Conclusion

In summary, we conclude that WB-EMS may be a viable option for those unable to engage in conventional exercise, although significant effects on cardiometabolic outcomes and more specifically, the MetS, should not necessarily be expected.

## Data Availability

The raw data supporting the conclusions of this article will be made available by the authors without undue reservation.

## Footnotes

^1^It is important to note that these adjuvant movements *per se* are not intended to generate musculoskeletal or cardiometabolic effects (i.e. non-superimposed concept of WB-EMS).

^2^However, due to the situation that only participants with the MetS were included, negative changes were not possible.

^3^NCEP-ATP II: National Cholesterol Education Program (NCEP) Expert Panel on Detection, Evaluation, And Treatment of High Blood Cholesterol in Adults (Adult Treatment Panel III).

^4^When comparing the HIIT+WB-EMS (*n* = 19) with the isolated HIIT group (*n* = 18) to determine the isolated effect of WB-EMS, no significant effects were observed.

^5^The authors reported non-significantly more favorable effects (*p* = 0.096) on the MetS-Z score (IDF-based) for the WB-EMS (*n* = 23) compared with the HIT-RT group (*n* = 23).

^6^This refers in particular to the comparisons with exercising control groups.
